# Reconsidering N component of cancer staging for T1-2N0-2M0 small-cell lung cancer: a retrospective study based on multicenter cohort

**DOI:** 10.1186/s12931-023-02440-3

**Published:** 2023-06-23

**Authors:** Lei-Lei Wu, Li-Hong Qiu, Xiaolu Chen, Wan-Jun Yu, Chong-Wu Li, Jia-Yi Qian, Shen-Hua Liang, Peng Lin, Hao Long, Lan-Jun Zhang, Zhi-Xin Li, Kun Li, Feng Jiang, Guo-Wei Ma, Dong Xie

**Affiliations:** 1 Department of Thoracic Surgery, Shanghai Pulmonary Hospital, School of Medicine, Tongji University, Shanghai, 200433 P. R. China; 2 Department of Thoracic Surgery, State Key Laboratory of Oncology in South China, Collaborative Innovation Center for Cancer Medicine, Sun Yat-sen University Cancer Center, Guangzhou, 510000 P. R. China; 3 Department of Respiratory and Critical Care, The Affiliated People’s Hospital of Ningbo University, Ningbo, 315100 P. R. China; 4 Department of Oncology, Zhongda Hospital, Southeast University, Nanjing, 210009 P. R. China

**Keywords:** Small cell lung cancer, T1-2N0-2M0, Nodal classification, New nodal classification, Multicenter database

## Abstract

**Background:**

The current nodal (pN) classification still has limitations in stratifying the prognosis of small cell lung cancer (SCLC) patients with pathological classifications T1-2N0-2M0. Thus. This study aimed to develop and validate a modified nodal classification based on a multicenter cohort.

**Materials and methods:**

We collected 1156 SCLC patients with pathological classifications T1-2N0-2M0 from the Surveillance, Epidemiology, and End Results database and a multicenter database in China. The X-tile software was conducted to determine the optimal cutoff points of the number of examined lymph nodes (ELNs) and lymph node ratio (LNR). The Kaplan-Meier method, the Log-rank test, and the Cox regression method were used in this study. We classified patients into three pathological N modification categories, new pN#1 (pN0-#ELNs > 3), new pN#2 (pN0-#ELNs ≤ 3 or pN1-2-#LNR ≤ 0.14), and new pN#3 (N1-2-#LNR > 0.14). The Akaike information criterion (AIC), Bayesian Information Criterion, and Concordance index (C-index) were used to compare the prognostic, predictive ability between the current pN classification and the new pN component.

**Results:**

The new pN classification had a satisfactory effect on survival curves (Log-rank *P* < 0.001). After adjusting for other confounders, the new pN classification could be an independent prognostic indicator. Besides, the new pN component had a much more accurate predictive ability in the prognostic assessment for SCLC patients of pathological classifications T1-2N0-2M0 compared with the current pN classification in the SEER database (AIC: 4705.544 vs. 4731.775; C-index: 0.654 vs. 0.617, *P* < 0.001). Those results were validated in the MCDB from China.

**Conclusions:**

The multicenter cohort developed and validated a modified nodal classification for SCLC patients with pathological category T1-2N0-2M0 after surgery. Besides, we propose that an adequate lymph node dissection is essential; surgeons should perform and consider the situation of ELNs and LNR when they evaluate postoperative prognoses of SCLC patients.

**Supplementary Information:**

The online version contains supplementary material available at 10.1186/s12931-023-02440-3.

## Introduction

Lung cancer is the first-rank cause of cancer-related mortality worldwide, and it mainly includes two histological types, non-small cell lung cancer and small cell lung cancer (SCLC) [1]. Most lung cancer is non-small cell lung cancer, which accounts for over 83.0%, and its 5-year overall survival rate reaches about 23% [2]. SCLC has a much worse survival than non-small cell lung cancer, of which the 5-year overall survival rate is approximately 6%, although it is only a small part of lung cancer (about 15%) [2]. As an important treatment approach, surgery provides survival benefits for limited-stage SCLC patients, confirmed by previous studies and recommended for patients with clinical classification T1-2N0M0 [3–5]. Thus, limited-stage SCLC patients with lung resection have better survival outcomes than those without surgery [3, 4, 6]. However, the prognosis of those patients remains heterogeneous [1, 7]. Differences in patient prognosis may imply different treatment strategies; therefore, it is important to assess the patient’s prognosis accurately. The tumor, nodal, and metastasis (TNM) classification system is the leading evaluation approach to predicting the prognosis [8]. The current 8th edition TNM category system was launched in 2017, which had a more accurate predictive ability to assess the patient prognosis than the previous edition [9]. However, the pathological nodal (pN) classification is still the same in those two editions [10]. The status of lymph nodes indicates whether SCLC patients with a clinical classification of T1-2N0M0 receive adjuvant radiotherapy [5]. Accordingly, accurate lymph node status estimates are the key to providing prognostic information and determining the appropriate therapy strategies.

However, the current pN classification still has some drawbacks and is limited by the level of lymph node dissection. Some studies suggested that the number of examined lymph nodes (ELNs) could affect patient survival [11, 12]. In addition, the status of pN classification does not reflect the tumor burden in the lymph node enough. Some researchers confirmed that lymph node ratio (LNR) might be a more accurate tool than the current pT classification to predict prognosis and guide the plan for adjuvant therapy [13–15]. As a consequence, ELNs and LNR may play important roles in the modification of pN classification.

Previous studies have assessed patient prognosis by ELN or LNR; however, the ELN or LNR usually appears alone [16–18]. For example, in one report, the LNR was primarily evaluated as an indication of patient prognosis, but the harm caused by insufficient lymph node dissections was not reflected. Although another study evaluated both ELN and LNR, this study included an analysis of patients with metastases of lymph nodes and did not include SCLC [15]. Also, most studies utilized publicly available data sets and did not validate each other with data from their hospitals [15–17].

Moreover, there is a lack of studies including ELNs and LNR in SCLC patients with pathological classification T1-2N0-2. Therefore, in the present study, we analyzed the cutoff points for the number of ELNs and LNR in SCLC patients with pathological classification T1-2N0-2M0 using data from the multicenter database (MCDB) in China (included four hospitals) and Surveillance, Epidemiology, and End Results (SEER) database. In addition, we further compared predictive ability between the current pN classification and a new pN classification that combined ELNs with LNR in patient prognosis. This study aimed to elucidate which pathological nodal classification is the best: the current pN classification or the new pN classification that combined ELNs with LNR.

## Materials and methods

### Patient selection

**Fig. 1:**
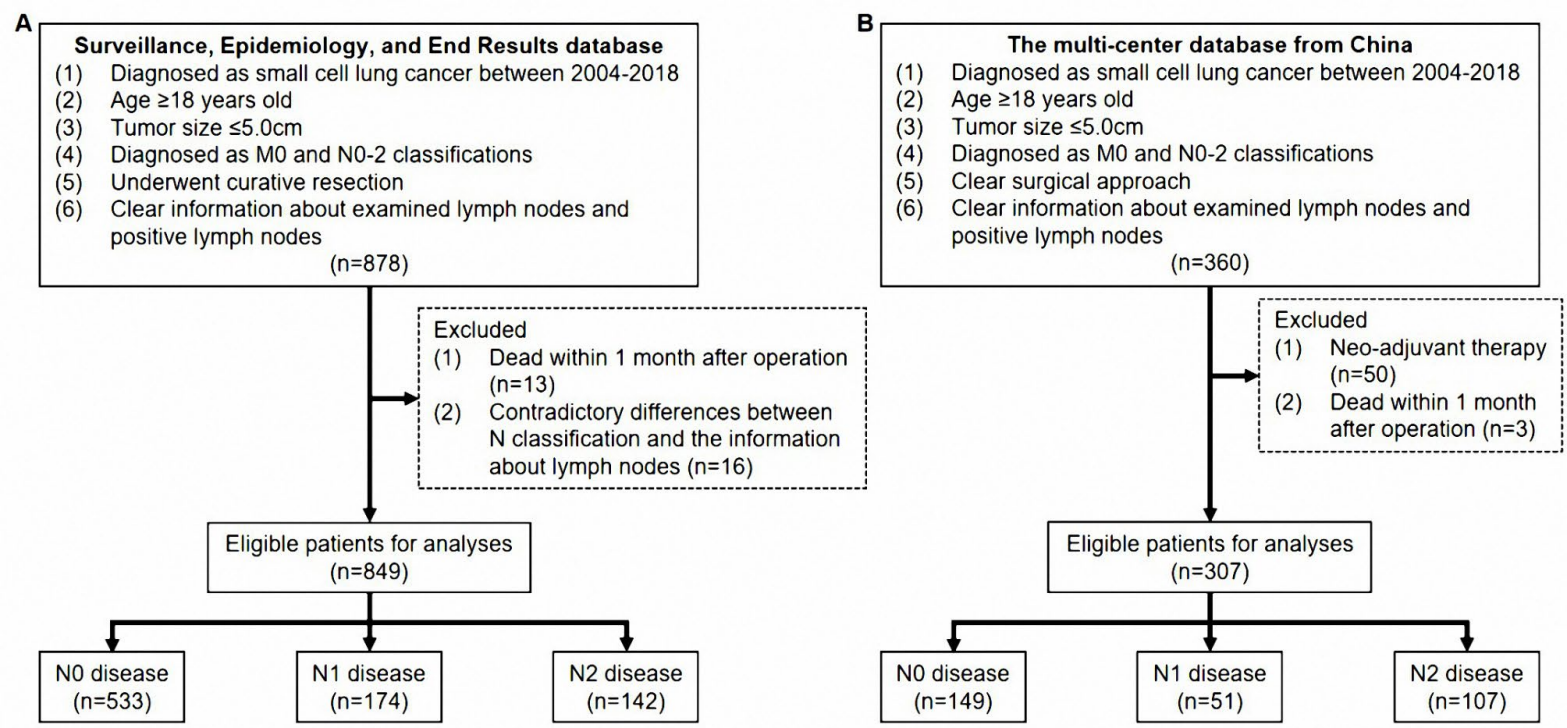
The standards of case selection in the SEER database (A) and MCDB from China (B)

A total of 1,156 SCLC patients with pathological classification T1-2N0-2M0 diagnosed between 2004 and 2018 were included in the present study. Eight hundred forty-nine cases were selected from the SEER database by SEER*Stat software (version 8.4.0), and 307 cases were collected from four hospitals in China (Shanghai Pulmonary Hospital, Sun Yat-sen University Cancer Center, Zhongda Hospital, and The Affiliated People’s Hospital of Ningbo University). Eligible patients for main analyses met the following criteria: (1) pathologically diagnosed as SCLC; (2) age ≥ 18 years old; (3) tumor size ≤ 5.0 cm; (4) diagnosed as classification M0 and N0-2; (5) known surgical approach; (6) known information about ELNs and positive lymph nodes. Patients were excluded if they: (1) dead within one month after surgery; (2) pN classification and lymph node records were inconsistent. Detailed information about patient selection standards is shown in Fig. 1. The work has been reported in line with the STROCSS criteria [19].

### Follow-up and data collection

The follow-up information on the SEER database and MCDB cohort from China was updated in November 2021 and March 2022, respectively. We used telephone and outpatient visit records for follow-up updates. The median follow-up time was 44.0 months (from 2 to 151 months). Patients after the operation visited the outpatient clinic at 3- or 6-month intervals for the first three years and 12-month intervals after that. The time interval between the diagnosis of the SCLC and the cancer-caused mortality was defined as cancer-specific survival (CSS). Cases were censored at the end of the follow-up. CSS was considered best concerning clinical relevance.

The patients’ clinical-pathological information was obtained from the SEER database: sex, age, tumor location, tumor grade, tumor size, surgical approaches, the number of ELNs, positive lymph nodes, radiotherapy record, chemotherapy record, and pN classification.

### Statistical analyses

The X-tile software was used to determine the optimal cutoff points of ELNs and LNR in the pN0-2 cohort and pN1-2 cohort, respectively [20]. X-tile was tested statistically by enumeration, using different values as the truncation value groups, and the test result with the smallest p-value was recognized as the best truncation value for the data set. According to the results of the X-tile software, we finally classified patients into three new pathological N categories, new pN#1 (pN0-#ELNs > 3), new pN#2 (pN0-#ELNs ≤ 3 or pN1-2-#LNR ≤ 0.14), and new pN#3 (N1-2-#LNR > 0.14). Survival curves were generated through the Kaplan-Meier method and compared by the Log-rank test. The nomograms were conducted to show the weight of all variables visually. Univariable and multivariable Cox regression analyses were performed to calculate the hazard ratio (HR) and 95% confidence interval (CI) of the variables for cancer-specific mortality. A two-sided *P* < 0.05 was defined as statistically significant. The − 2LogLikelihood value, Akaike information criterion (AIC), and Bayesian Information Criterion (BIC) were calculated according to Cox regression. The abovementioned three indicators mean a more accurate model when their values are smaller [21]. The concordance index (C-index) and the area under the receiver operating characteristic (ROC) curve (AUC) were used to compare the prognostic, predictive ability between the current pN classification and N modification. The standard error (SE) was performed to evaluate the stability of the C-index. C-index and SE were calculated by “compareC” packages and nomograms constructed by “survival” and “rms” packages using R 4.1.2 software (https://www.r-project.org/), then other analyses were performed using software SPSS 25.0 (IBM SPSS, Inc., Armonk, IL, USA).

## Results

### Patient characteristics

In the SEER database, 849 patients with a median survival time of 31.0 months (range 1-190 months) were identified as 533 pN0 cases (62.8%), 174 pN1 cases (20.5%), and 142 pN2 cases (16.7%). Females outnumbered men, constituting 56.5% of the patients. 369 (43.5%) patients were aged 65 and below, whereas 480 (56.5%) were over 65 years old. The optimal cutoff value of ELNs in the entire cohort was 3. Thus, 23.0% and 77.0% of patients were categorized into the ELNs ≤ 3 and ELNs > 3 (**Supplementary Fig. 1**), respectively. Of note, 89 patients with ELNs ≤ 3 were in the cohort with lobectomy/ pneumonectomy; 88 patients with ELNs > 3 were in the cases with sub-lobectomy. LNR’s optimal cutoff point was 0.14 in the cohort with pN1-2 (**Supplementary Fig. 2**). Therefore, pN1-2 patients with LNR ≤ 0.14 and pN0 patients were combined into one group (n = 628, 74.0%), and patients with LNR > 0.14 were classified into another group (n = 221, 26.0%). According to the prognostic performance of ELNs and LNR, we combined those two factors to develop a new indicator, N modification. Overall, 402, 226, and 221 cases were classified as new pN#1 (pN0-#ELNs > 3), new pN#2 (pN0-#ELNs ≤ 3 or pN1-2-#LNR ≤ 0.14), and new pN#3 (N1-2-#LNR > 0.14), respectively.

As for MCDB from China, 307 cases with a median survival time of 33.0 months (range 2-152 months) included 149 pN0 diseases (48.5%), 51 pN1 diseases (16.6%), and 107 pN2 diseases (34.9%), respectively. There were 19 patients with ELNs > 3 in the group of sub-lobectomy and 23 patients with ELNs ≤ 3 in the category of lobectomy/ pneumonectomy. Overall, 30.4%, 28.0%, and 31.6% of cases were classified as N modification 1, N modification 2, and N modification 3. The patients’ other baseline characteristics are presented in Table 1.

**Table 1:** The characteristics of small cell lung cancer patients

		SEER	MCDB
Total		n = 849	n = 307
Sex	Male	369 (43.5%)	268 (87.3%)
	Female	480 (56.5%)	39 (12.7%)
Age	≤ 65	369 (43.5%)	182 (59.3%)
	> 65	480 (56.5%)	125 (40.7%)
Surgery	Lobectomy/ pneumonectomy	655 (77.1%)	277 (90.2%)
	Sub-lobectomy	194 (22.9%)	30 (9.8%)
Radiotherapy	None	545 (64.2%)	263 (85.7%)
	Yes	289 (34.0%)	44 (14.3%)
	Unknown	15 (1.8%)	0 (0%)
Chemotherapy	None	273 (32.2%)	100 (32.6%)
	Yes	576 (67.8%)	207 (67.4%)
Location	Upper lobe	510 (60.1%)	170 (55.4%)
	Lower lobe	263 (31.0%)	110 (35.8%)
	Other/ unknown	76 (9.0%)	27 (8.8%)
Grade	I-II	47 (5.5%)	0 (0%)
	III	312 (36.7%)	82 (26.7%)
	IV	249 (29.3%)	66 (21.5%)
	Unknown	241 (28.5)	159 (51.8)
Examined lymph nodes	≤ 3	195 (23.0%)	34 (11.1%)
	> 3	654 (77.0%)	273 (88.9%)
Tumor size	≤ 3.0 cm	649 (76.4%)	192 (62.5%)
	> 3.0 cm	200 (23.6%)	115 (37.5%)
pN classification	pN0	533 (62.8%)	149 (48.5%)
	pN1	174 (20.5%)	51 (16.6%)
	pN2	142 (16.7%)	107 (34.9%)
Lymph node ratio	≤ 0.14	628 (74.0%)	209 (68.1%)
	> 0.14	221 (26.0%)	98 (31.9%)
New pN classification	New pN#1	402 (47.3%)	124 (40.4%)
	New pN#2	226 (26.6%)	86 (28.0%)
	New pN#3	221 (26.1%)	97 (31.6%)

SEER: Surveillance, Epidemiology, and End Results, MCDB: multi-center database; pN: pathological nodal

### Survival effect of ELNs and LNR

**Fig. 2:**
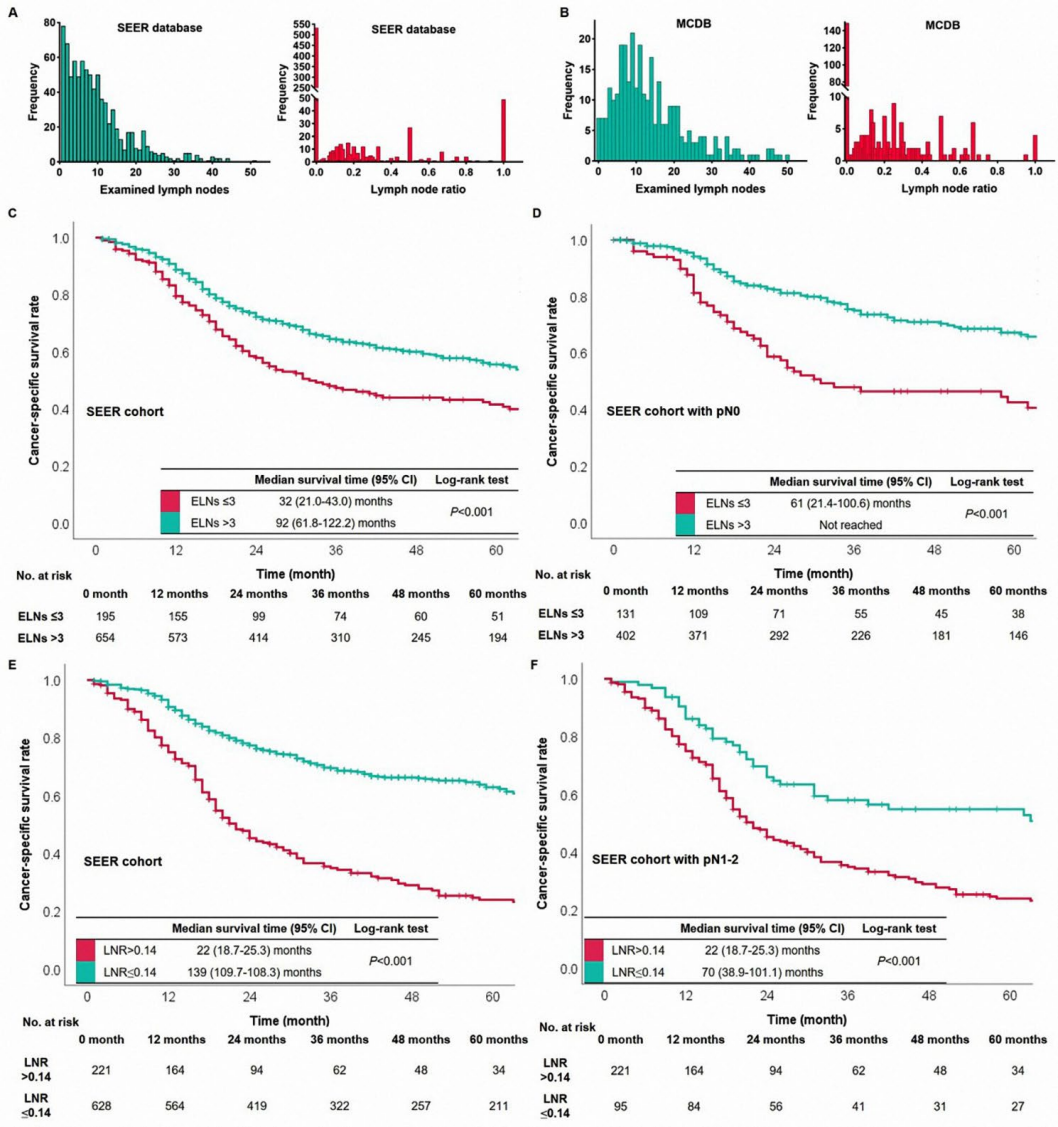
The distribution of ELNs and LNR in the SEER database (A) and MCDB from China (B). The survival curves based on ELNs were in the SEER database (C) and the pN0 patients of SEER database (D). The survival curves based on LNR were in the SEER database (E) and the pN1-2 patients of SEER database (F)

**Fig. 3:**
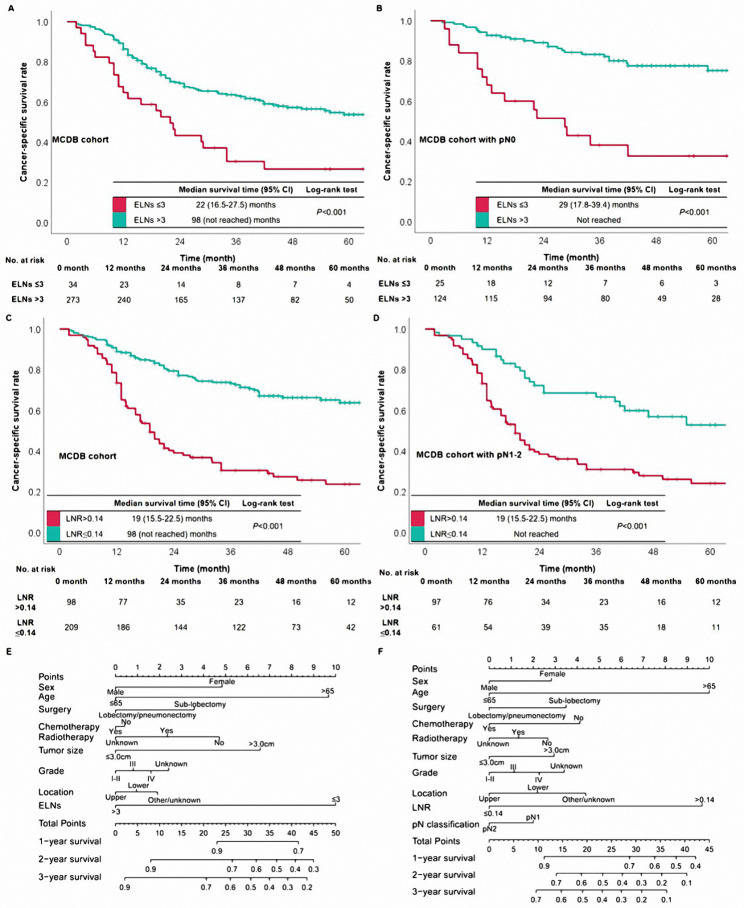
The survival curves based on ELNs were in the MCDB (A) and the pN0 patients of MCDB (B). The survival curves based on LNR were in the MCDB (C) and the pN1-2 patients of MCDB (D). The nomograms of the pN0 patients (E) and the pN1-2 patients (F) were constructed in the SEER database

The median values of ELNs were 8 (range 1–51) and 11 (range 0–50) in the SEER database and MCDB from China, respectively. LNR’s median values were 0 and 0.04 in the SEER database and MCDB, respectively. The distribution of ELNs and LNR in the abovementioned database is shown in Fig. 2A-B. Patients’ survival curves were stratified successfully according to the optimal points of ELNs and LNR generated from the SEER database (Fig. 2C-F). Besides, the same cutoff points also performed prognostic significance in the MCDB cohort (Fig. 3A-D). In short, patients with ELNs > 3 or LNR ≤ 0.14 had s better survival than those with ELNs ≤ 3 or LNR > 0.14.

**Fig. 4:**
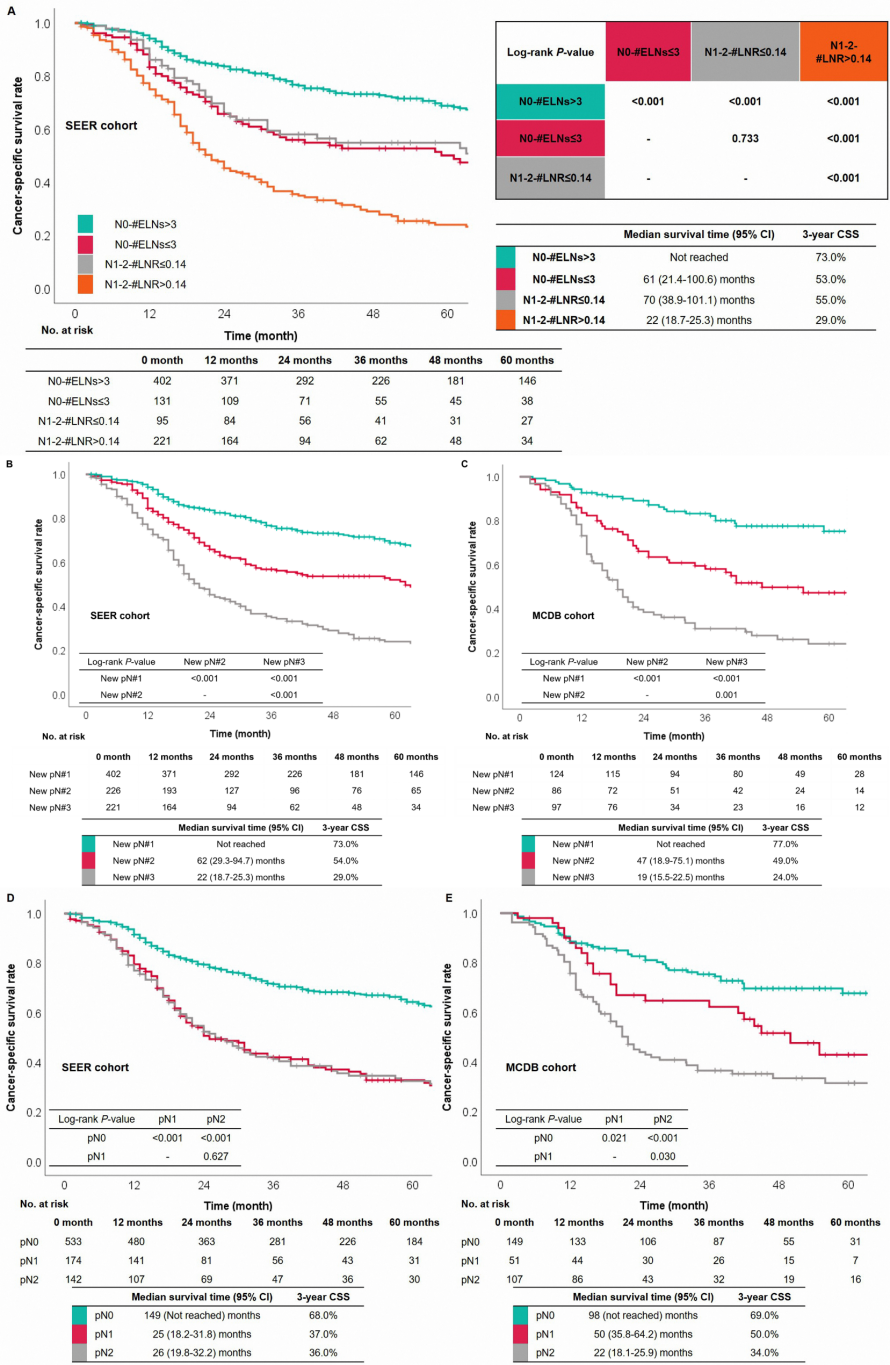
The survival curves were based on ELNs and LNR (A). The survival curves based on the N modification were in the SEER database (B) and the MCDB from China (C). The survival curves based on the current pN modification were in the SEER database (D) and the MCDB from China (E)

Then, the nomograms were constructed to visually evaluate the weight of ELNs and LNR to affect survival in the pN0 cohort and the pN1-2 cohort in the SEER database, respectively (Fig. 3E-F). In the nomogram of the pN0 cohort, ELNs play the most important role in affecting survival; LNR’s weight was larger than the pN classification in the pN1-2 cohort. Therefore, we classified patients into four categories, N0-#ELNs > 3, N0-#ELNs ≤ 3, N1-2-#LNR ≤ 0.14, and N1-2-#LNR > 0.14. The survival curves based on the abovementioned four categories were drawn (Fig. 4A). The prognosis of N0-#ELNs ≤ 3 patients was similar to those with N1-2-#LNR ≤ 0.14 (Log-rank; *P* = 0.733). To further develop a new pN classification, we combined cases with N0-#ELNs ≤ 3 with those with N1-2-#LNR ≤ 0.14.

### Prognostic significance of N modification

The new pN category included new pN#1 (pN0-#ELNs > 3), new pN#2 (pN0-#ELNs ≤ 3 or pN1-2-#LNR ≤ 0.14), and new pN#3 (N1-2-#LNR > 0.14). Overall, patients with new pN#1 had the best survival than the other two classifications (Fig. 4B). The 3-year CSS rates were 73.0%, 54.0%, and 29.0% in the cases of new pN#1, new pN#2, and new pN#3, respectively. The multivariable Cox regression identified the new pN classification as an independent prognostic indicator (all *P* < 0.001, Table 2). In addition, there was descreasing survival trend by category of the N modification (all *P* < 0.001, Fig. 4B).

**Table 2:** Univariable and multivariable analyses for cancer-specific mortality in the SEER database

	Univariable analysis			Multivariable analysis			
Variable	HR (95% Cl)	*P*-value		HR 95%Cl	*P*-value	HR (95%CI)	*P*-value
**Sex**							
Male	1 [reference]			1 [reference]		1 [reference]	
Female	0.80 (0.65–0.97)	0.025		0.79 (0.66–0.96)	0.019	0.77 (0.63–0.94)	0.011
**Age (years)**							
≤ 65	1 [reference]			1 [reference]		1 [reference]	
> 65	1.79 (1.45–2.20)	< 0.001		1.88 (1.51–2.33)	< 0.001	1.88 (1.51–2.33)	< 0.001
**Location**							
Upper lobe	1 [reference]			1 [reference]		1 [reference]	
Lower lobe	1.29 (1.04–1.60)	0.019		1.23 (0.99–1.53)	0.062	1.19 (0.96–1.48)	0.113
Other/unknown	1.23 (0.88–1.72)	0.230		1.27 (0.90–1.78)	0.174	1.16 (0.83–1.63)	0.390
**Tumor size**							
≤ 3.0 cm	1 [reference]			1 [reference]		1 [reference]	
> 3.0 cm	1.30 (1.04–1.62)	0.022		1.36 (1.08–1.71)	0.010	1.34 (1.07–1.69)	0.012
**Grade**							
I-II	1 [reference]			1 [reference]		1 [reference]	
III	1.08 (0.69–1.69)	0.743		1.37 (0.86–2.16)	0.181	1.33 (0.84–2.11)	0.218
IV	1.19 (0.75–1.87)	0.460		1.43 (0.90–2.26)	0.130	1.37 (0.87–2.17)	0.174
Unknown	1.17 (0.74–1.85)	0.508		1.48 (0.92–2.36)	0.103	1.43 (0.90–2.28)	0.136
**Surgery Approach**							
Lobectomy/pneumonectomy	1 [reference]			1 [reference]		1 [reference]	
Sub-lobectomy	1.53 (1.22–1.92)	< 0.001		1.50 (1.18–1.90)	0.001	1.22 (0.97–1.55)	0.093
**Chemotherapy**							
No	1 [reference]			1 [reference]		1	
Yes	1.01 (0.81–1.25)	0.954		0.77 (0.60-1.00)	0.046	0.86 (0.67–1.11)	0.250
**Radiotherapy**							
No	1 [reference]			1 [reference]		1 [reference]	
Yes	1.26 (1.03–1.55)	0.024		1.01 (0.79–1.29)	0.957	0.93 (0.73–1.19)	0.578
Unknown	0.79 (0.35–1.78)	0.566		0.58 (0.25–1.34)	0.202	0.55 (0.24–1.25)	0.152
**pN classification**							
pN0	1 [reference]			1 [reference]			
pN1	2.45 (1.93–3.11)	< 0.001		2.88 (2.24–3.72)	< 0.001		
pN2	2.64 (2.06–3.37)	< 0.001		2.75 (2.09–3.61)	< 0.001		
**New pN classification**							
New pN#1	1 [reference]					1 [reference]	
New pN#2	2.01 (1.55–2.60)	< 0.001				1.94 (1.49–2.53)	< 0.001
New pN#3	3.76 (2.96–4.76)	< 0.001				4.07 (3.14–5.28)	< 0.001
**-2LogLikelihood value**				4705.775		4677.544	
**AIC**				4731.775		4705.544	
**BIC**				4800.192		4771.961	
** C-index (SE)**				0.617 (0.013)		0.654 (0.014)	

SEER: Surveillance, Epidemiology, and End Results, HR: hazard ratio, CI: confidence interval, pN: pathological node, AIC: Akaike information criterion, BIC: Bayesian Information Criterion, C-index: concordance index, SE: standard error. The method of Cox regression was “Enter selection”

To further validate the performance of the new pN classification, we used the same classification in the MCDB cohort. Similarly, in the MCDB cohort, patients’ prognoses showed a decreasing trend by the new pN classification (all *P* < 0.001, Fig. 4C). The median survival time of patients with the new pN#1, new pN#2, and new pN#3 were 98 months, 50 months, and 22 months, respectively. Besides, the new pN classification was confirmed as an independent prognosticator (Table 3).

**Table 3:** Univariable and multivariable analyses for cancer-specific mortality in the multi-center database from China

	Univariable analysis			Multivariable analysis			
Variable	HR (95% Cl)	*P*-value		HR 95%Cl	*P*-value	HR (95%CI)	*P*-value
**Sex**							
Male	1 [reference]			1 [reference]		1 [reference]	
Female	0.87 (0.51–1.48)	0.600		1.13 (0.66–1.95)	0.651	1.12 (0.65–1.93)	0.693
**Age (years)**							
≤ 65	1 [reference]			1 [reference]		1 [reference]	
> 65	1.33 (0.95–1.87)	0.096		1.28 (0.90–1.82)	0.163	1.27 (0.89–1.81)	0.183
**Location**							
Upper lobe	1 [reference]			1 [reference]		1 [reference]	
Lower lobe	0.94 (0.66–1.36)	0.952		1.19 (0.81–1.75)	0.377	1.05 (0.71–1.54)	0.822
Other/unknown	1.14 (0.64–2.02)	0.657		1.10 (0.61–1.99)	0.745	0.96 (0.53–1.74)	0.900
**Tumor size**							
≤ 3.0 cm	1 [reference]			1 [reference]		1 [reference]	
> 3.0 cm	1.23 (0.88–1.73)	0.234		1.10 (0.77–1.56)	0.597	1.11 (0.77–1.58)	0.582
**Grade**							
III	1 [reference]			1 [reference]		1 [reference]	
IV	2.53 (1.59–4.04)	< 0.001		2.97 (1.83–4.84)	< 0.001	3.03 (1.85–4.97)	< 0.001
Unknown	1.10 (0.71–1.70)	0.667		1.46 (0.91–2.34)	0.119	1.37 (0.86–2.19)	0.183
**Surgery Approach**							
Lobectomy/pneumonectomy	1 [reference]			1 [reference]		1 [reference]	
Sub-lobectomy	0.97 (0.55–1.72)	0.918		0.98 (0.54–1.77)	0.943	0.94 (0.52–1.70)	0.832
**Chemotherapy**							
No	1 [reference]			1 [reference]		1	
Yes	0.82 (0.58–1.16)	0.265		0.72 (0.49–1.06)	0.093	0.69 (0.47–1.01)	0.053
**Radiotherapy**							
No	1 [reference]			1 [reference]		1 [reference]	
Yes	1.02 (0.64–1.63)	0.932		0.72 (0.44–1.19)	0.198	0.68 (0.41–1.12)	0.131
**pN classification**							
pN0	1 [reference]			1 [reference]			
pN1	1.77 (1.07–2.91)	0.025		2.10 (1.26–3.50)	0.005		
pN2	2.93 (2.00-4.29)	< 0.001		3.46 (2.30–5.22)	< 0.001		
**New pN classification**							
New pN#1	1 [reference]					1 [reference]	
New pN#2	2.59 (1.59–4.21)	< 0.001				3.06 (1.85–5.05)	< 0.001
New pN#3	5.01 (3.20–7.85)	< 0.001				6.06 (3.78–9.71)	< 0.001
**-2LogLikelihood value**				1380.058		1352.247	
**AIC**				1404.058		1376.247	
**BIC**				1448.780		1420.969	
** C-index (SE)**				0.622 (0.041)		0.679 (0.035)	

HR: hazard ratio, CI: confidence interval, pN: pathological node, AIC: Akaike information criterion, BIC: Bayesian Information Criterion, C-index: concordance index, SE: standard error. The method of Cox regression was “Enter selection”

### Comparison between the new pN and the current pN classifications

Survival curves based on the current pN classification were drawn in order to compare further the predictive performance between the new pN and current pN classifications. The stratified effect of survival curves originating from the current pN classification was not as distinct as those derived from the new pN category (Fig. 4D-E). The values of -2LogLikelyhood, AIC, and BIC were smaller in the multivariable Cox regression model that included the new pN category than those in the model that included the current pN classification (Tables 2 and 3). Thus, the new pN classification had a better ability to indicate prognosis than the current pN classification.

**Fig. 5:**
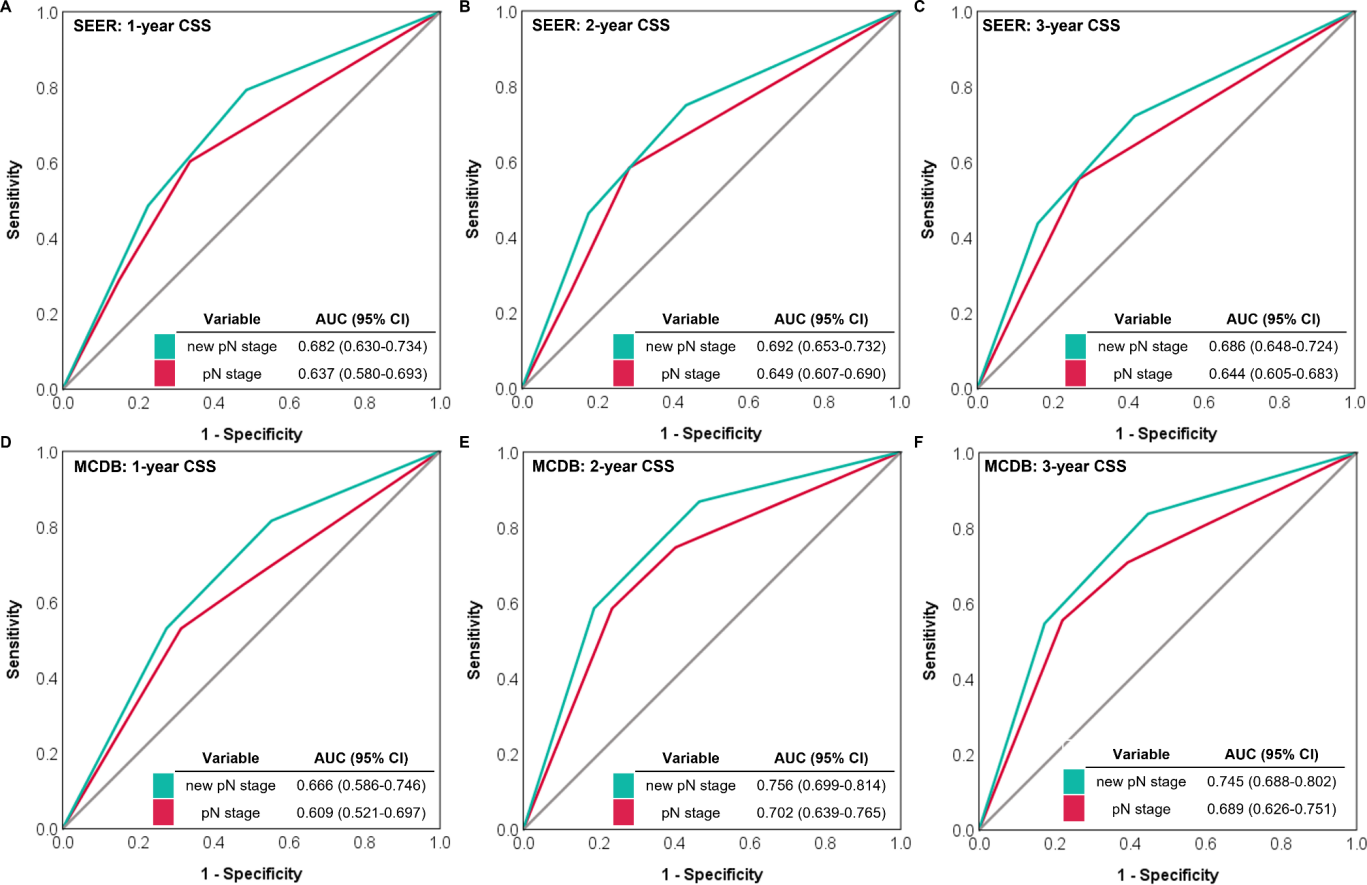
The ROCs of 1-year, 2-year, and 3-year CSS were in the SEER database (A-C) and the MCDB from China (D-F).

Besides, the C-index was also calculated to compare the predictive effect between the current pN and the new pN classifications (SEER: 0.617 vs. 0.654, *P* < 0.001, Table 2; MCDB: 0.622 vs. 0.679, *P* < 0.001, Table 3). We further drew the ROC curves and calculated the AUC to validate the performance of the new pN category. The results of the analysis for the SEER database showed that the new pN classification had higher values of AUC in predicting 1-year CSS (0.682 vs. 0.637), 2-year CSS (0.692 vs. 0.649), and 3-year CSS (0.686 vs. 0.644) than the current pN classification, respectively. Similar results were also presented in the analysis of the MCDB cohort (Fig. 5).

## Discussion

SCLC is a small part of lung cancer that has received less attention from investigators than non-small cell lung cancer. In thoracic surgical oncology, surgeons seem more inclined to discuss which combined stages of the disease are appropriate to undergo surgical resection. The National Comprehensive Cancer Network guidelines recommend that SCLC patients with clinical stage I-IIA (classification T1-2N0M0) perform a surgical operation [5]. Thus, recent studies on the surgical treatment of SCLC have explored the role of surgery in stage III patients [3, 6, 22, 23]. Based on their results, surgery was considered a potential treatment for stage III SCLC patients, which provided patients with an optional therapeutic approach, though their research results were not consistent enough. However, there are few studies on lymph node dissection and pN classification in the limited-stage SCLC patients. The current pN classification was based on the idea that lymph node metastasis follows the direction of lymphatic drainage from intrapulmonary lymph nodes (pN1) to mediastinal lymph nodes (pN2) and then to distant lymph nodes (pN3). However, in some studies and our clinical practice, it has been found that lymph node metastases can skip the N1 station and jump directly to the N2 station (skip-N2). Moreover, patients with the N1 station and N2 station metastases are also classified as N2. However, although both are N2, the prognosis of patients with skip-N2 metastases is much better than those with N2 metastases that include N1 stations [24–26]. In other words, the current pN classification is likely to have some flaws in evaluating the prognosis of SCLC patients after surgery. Accordingly, some researchers used LNR and the number of positive lymph nodes to supplement the pN classification in order to strengthen the predictive ability [15, 27]. Regrettably, both of their studies only collected non-small cell lung cancer patients. Therefore, to the best of our knowledge, there is still information lacking regarding improving pN classification in SCLC.

In the present study, we used a large cohort from the SEER and the multicenter database in China to develop and validate a new pathological nodal classification. ELNs and LNR reflected the status of lymph node dissection and lymph node metastasis and reached optimal cutoff points by X-tile software. We develop a new pN classification. The new pN classification had a better predictive ability than the current pN classification. Therefore, we propose that the new pN classification that combines LNR and ELNs has a better performance than the current pN classification in predicting SCLC patients’ prognoses.

A sufficient lymph node dissection is key to categorizing the pathological nodal classification and evaluating the prognosis precisely. Previous studies confirmed that an insufficient lymph node dissection was associated with a poor prognosis in the field of non-small cell lung cancer [12, 16]. Besides, the survival benefit of lobectomy was unable to be accurately assessed because of lack of lymph node dissection information compared with sub-lobectomy, according to a recent report [28]. The studies mentioned above show that adequate lymph node dissection plays a vital role in thoracic surgical oncology. Nevertheless, the current guidelines about SCLC do not indicate a consistent number of lymph nodes necessary [5]. Analysis of data from 1051 limited-stage SCLC patients indicated that survival was improved s when the ELNs were over 7 [29]. The threshold for the number of ELNs from their study was more extensive than ours. The reason might be that they excluded patients with sub-lobectomy; however, we did not. We found that patients with sub-lobectomy also performed lymph node dissection when we processed the data; therefore, we decided to reserve this part of the data. Besides, another study performed the same operation of data as the present study [17]. Regardless of the difference in the number of ELNs, all demonstrated similar results that inadequate lymph node dissections decreased the survival time of limited-stage SCLC patients after surgery.

LNR is a simple and effective tool for postoperative prognostic assessment for SCLC patients with classifications T1-2N0-2M0. However, it is undeniable that the calculation of LNR is still based on lymph node dissection. The present study showed that patients with N0-#ELNs ≤ 3 had similar survival to those with N1-2-#LNR ≤ 0.14. Those results might suggest that patients with inadequate lymph node dissection were accompanied by the diseases of lymph node metastasis. The low number of ELNs masked the actual metastatic status of the lymph nodes. Accordingly, the N modification that combined ELNs and LNR might reflect the actual nodal situation, which showed a better predictive ability than the current pN classification in the present study. Similarly, the study from *Li F at el.* also demonstrated that the pN classification combined with the number of metastatic lymph nodes or LNR had a more accurate performance than the current pN classification alone in the non-small cell lung cancer patients with pN1-2 classification [15]. On the one hand, these results illustrated the shortcomings of the current pN classification and, on the other hand, suggested that the combination of LNR improved the prognostic, predictive accuracy of the pN classification. Thus, we suggest that the next 9th edition TNM staging system should consider the information about lymph nodes except the anatomical location of lymph nodes, such as ELNs, LNR, and the number of metastatic lymph nodes.

There were still some limitations in this study. First, the present research was a retrospective study; therefore, some confounders were not included in the study; selection bias was still possible. Second, the treatment sequence of chemotherapy and radiotherapy in the SEER database was unknown. Thus, the cases with neoadjuvant therapy were not excluded from the SEER database. However, we excluded cases with neoadjuvant therapy in the multicenter database from China and reached similar results after analyses for the MCDB to validate the performance of the N modification. Third, the distribution of baseline characteristics still had some differences between the MCDB and SEER databases, though the sample size of MCDB was large. Finally, the N modification was confirmed to have a better performance than the current pN classification, but this does not mean it could replace the traditional TNM staging system. We need more studies to confirm our findings.

## Conclusions

In this multicenter cohort we developed and validated a new nodal classification for SCLC patients with pathological category T1-2N0-2M0 after surgery. Besides, we propose that an adequate lymph node dissection is essential; surgeons should perform and consider the situation of ELNs and LNR when they evaluate postoperative prognoses of SCLC patients.

## Electronic supplementary material

Below is the link to the electronic supplementary material.

Supplementary Material 1: The X-tile software determined the optimal cutoff point of ELNs. ELNs: examined lymph nodes

Supplementary Material 2: The X-tile software determined the optimal cutoff point of LNR. LNR: lymph node ratio

## Declarations

### Data statement

The data sets are available from the corresponding author upon reasonable request.

## Abbreviations

SCLC: Small cell lung cancer
TNM: Tumor, nodal and metastasis
pN: Pathological nodal
ELNs: Examined lymph nodes
LNR: Lymph node ratio
MCDB: Multicenter database
SEER: Surveillance, Epidemiology, and End Results
CSS: Cancer-specific survival
HR: Hazard ratio
CI: Confidence interval
AIC: Akaike information criterion
BIC: Bayesian Information Criterion
C-index: Concordance index
ROC: Receiver operating characteristic
AUC: Area under the ROC curve
SE: Standard error.
