# Supplementary material for: Reconsidering N component of cancer staging for T1-2N0-2M0 small-cell lung cancer: a retrospective study based on multicenter cohort
Source: Respir Res. 2023 Jun 23;24:168. doi: 10.1186/s12931-023-02440-3 (PMC10288722; doi:10.1186/s12931-023-02440-3)
Supplement: Supplementary file 1 — Supplementary Material 1: The X-tile software determined the optimal cutoff point of ELNs. ELNs: examined lymph nodes [file 12931_2023_2440_MOESM1_ESM.pdf]

Supplementary figure 1: The X-tile software determined the optimal cutoff point of ELNs. ELNs: examined lymph nodes

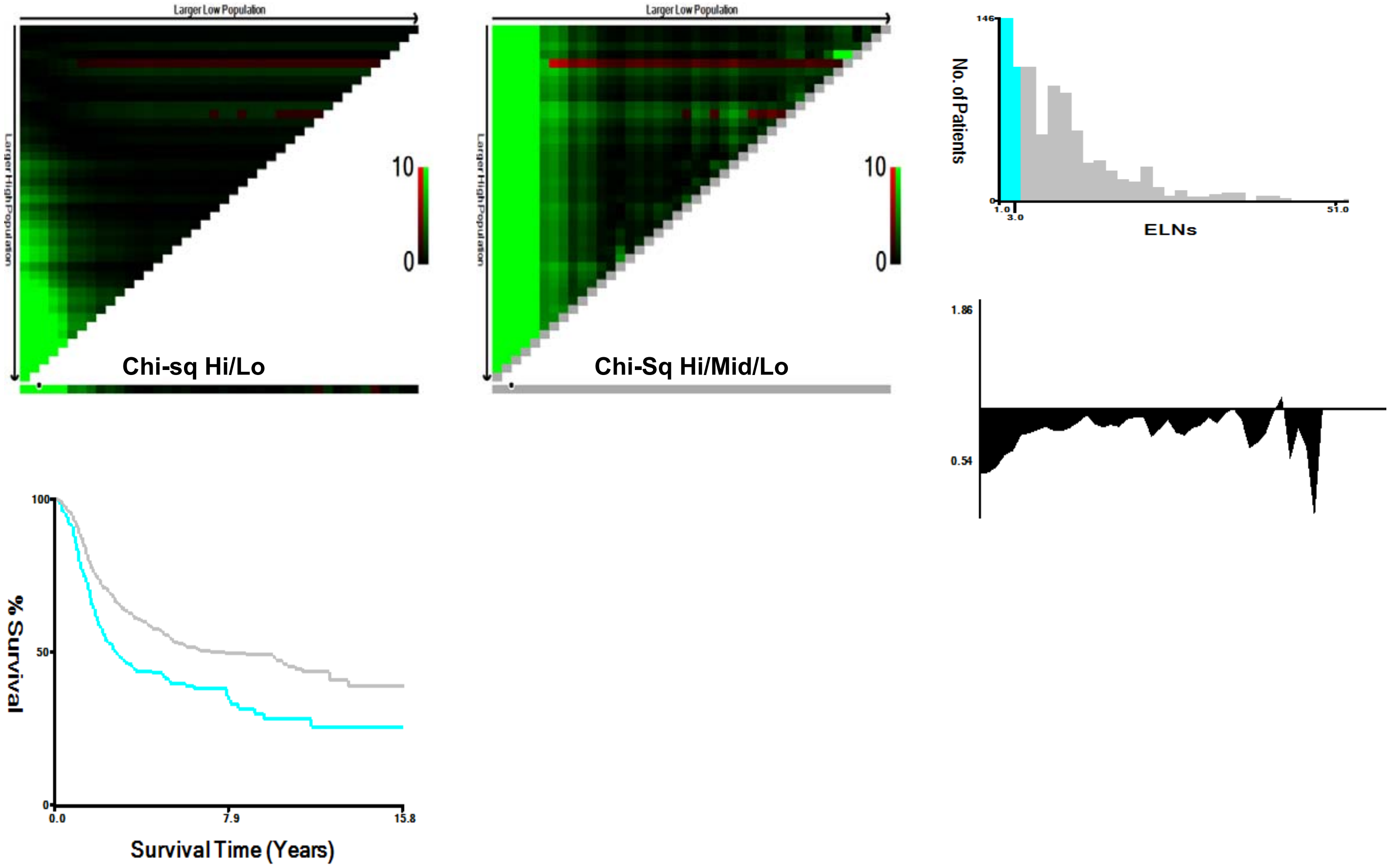

Subpopulation Cutpoints:

Pt No	% Total	Events	Rate	Rank	Range
195	22.97	114	58.46	0 to 2	1.00 thru 3.00
654	77.03	278	42.51	3 to 42	4.00 thru 51.00
849	100.00	392	46.17	0 to 42	1.00 thru 51.00

Statistics:

Variable	Value	
Miller-Seigmund P	0.0008	Max: 0.0008
Chi-sq Hi/Lo	18.0806	Max: 18.0806
Relative Risk 1 vs 2	1.38 / 1.00	

Copyright: Camp/Rimm, Yale University
www.tissuearray.org
