# Supplementary material for: Reconsidering N component of cancer staging for T1-2N0-2M0 small-cell lung cancer: a retrospective study based on multicenter cohort
Source: Respir Res. 2023 Jun 23;24:168. doi: 10.1186/s12931-023-02440-3 (PMC10288722; doi:10.1186/s12931-023-02440-3)
Supplement: Supplementary file 2 — Supplementary Material 2: The X-tile software determined the optimal cutoff point of LNR. LNR: lymph node ratio [file 12931_2023_2440_MOESM2_ESM.pdf]

Supplementary figure 2: The X-tile software determined the optimal cutoff point of LNR. LNR: lymph node ratio

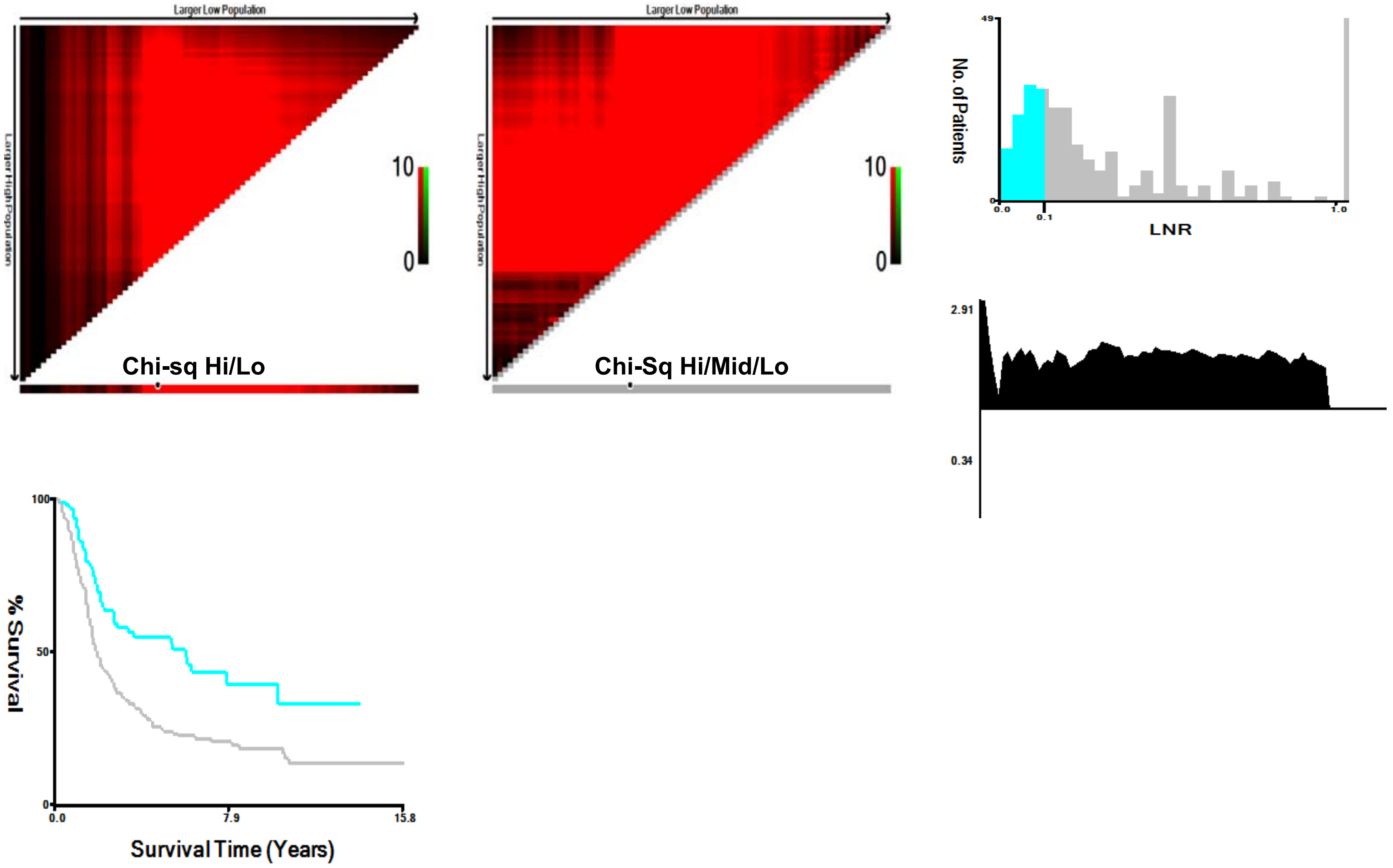

Subpopulation Cutpoints:

Pt No	% Total	Events	Rate	Rank	Range
95	30.06	45	47.37	0 to 27	0.02 thru 0.14
221	69.94	161	72.85	28 to 78	0.15 thru 1.00
316	100.00	206	65.19	0 to 78	0.02 thru 1.00

Statistics:

Variable	Value	
Miller-Seigmund P	0.0019	Max: 0.0019
Chi-sq Hi/Lo	16.4216	Max: 16.4216
Relative Risk 1 vs 2	1.00 / 1.54	

Copyright: Camp/Rimm, Yale University
www.tissuearray.org
